# Point-of-care testing in community pharmacies to improve antimicrobial stewardship in respiratory infections: a scoping review of effectiveness, implementation and cost-effectiveness—right-to-reply

**DOI:** 10.1093/jacamr/dlag217

**Published:** 2026-10-08

**Authors:** Sajal K Saha, Shukla Promite, Carly L Botheras, Elizabeth Manias, Nomvuyo Mothobi, Suzanne Robinson, Eugene Athan

**Affiliations:** Centre for Innovation in Infectious Disease and Immunology Research (CIIDIR), Institute for Mental and Physical Health and Clinical Translation (IMPACT), School of Medicine, Deakin University, Geelong, Victoria, Australia; National Centre for Antimicrobial Stewardship, The University of Melbourne, Melbourne, Victoria, Australia; Great Southern Bank, Werribee, Melbourne, Victoria, Australia; Centre for Innovation in Infectious Disease and Immunology Research (CIIDIR), Institute for Mental and Physical Health and Clinical Translation (IMPACT), School of Medicine, Deakin University, Geelong, Victoria, Australia; Monash Nursing and Midwifery, Faculty of Medicine, Nursing and Health Sciences, Monash University, Clayton, Victoria, Australia; AMR-One Health Working Group, Burnet Institute, Melbourne, Victoria, Australia; Deakin Health Economics, Institute for Health Transformation, Deakin University, Geelong, Victoria, Australia; EnAble Institute, Curtin University, Perth, Western Australia, Australia; Centre for Innovation in Infectious Disease and Immunology Research (CIIDIR), Institute for Mental and Physical Health and Clinical Translation (IMPACT), School of Medicine, Deakin University, Geelong, Victoria, Australia; Deakin Clinical Trials Hub, Deakin University, Geelong, Victoria, Australia

Dear Editor,

We thank the author for their thoughtful comments on our scoping review and for highlighting important considerations regarding the implementation of point-of-care testing (POCT) for respiratory tract infections in low- and middle-income countries (LMICs) and informal healthcare settings. We welcome the opportunity to further discuss these issues.

We agree that antimicrobial resistance (AMR) disproportionately affects LMICs and that implementation strategies for antimicrobial stewardship must be tailored to local health systems, workforce structures and regulatory environments.^1^ Indeed, one of the principal findings of our review was the limited geographical diversity of the available evidence. As reported, 26 of the 27 included studies were conducted in high-income countries, highlighting a substantial evidence gap and the need for implementation research in LMICs and other underrepresented settings. Accordingly, we recommended context-specific implementation strategies and further evaluation across diverse healthcare systems rather than assuming direct transferability of existing models.

The correspondence appropriately highlights the role of informal healthcare providers in many LMICs. We agree that, in parts of sub-Saharan Africa, South Asia and Southeast Asia,^2–4^ informal healthcare providers are often the first point of care, where non-prescription antibiotic supply remains widespread. However, our review was intentionally scoped to community pharmacy settings, reflecting both the available evidence and the objectives of the review. Consequently, our findings should not be interpreted as recommendations for implementation in informal medicine outlets or other unregulated healthcare settings. We agree that implementation strategies for these providers will require distinct behavioural, regulatory and health system approaches. This represents an important priority for future research.

We also agree that diagnostic stewardship is fundamental to antimicrobial stewardship. As highlighted in our review, successful implementation of POCT extends well beyond diagnostic accuracy and depends on workforce training, quality assurance, clinical governance, reimbursement mechanisms, workflow integration and patient acceptance. These implementation determinants were systematically mapped using the Consolidated Framework for Implementation Research, demonstrating that diagnostic technologies alone are insufficient to optimize antibiotic use without supportive implementation systems.

We acknowledge the landmark trials from Vietnam, Thailand and Myanmar demonstrating that C-reactive protein POCT can safely reduce unnecessary antibiotic prescribing in LMIC primary care.^5,6^ These studies provide encouraging evidence that POCT can be effective in LMIC settings when implemented within appropriate clinical and organizational infrastructure. Nevertheless, further implementation research is needed to determine how such interventions can be sustainably integrated into routine practice across different health systems, particularly where diagnostic infrastructure, regulatory oversight, workforce capability and financing mechanisms remain limited.

Finally, we agree that future evidence syntheses should examine POCT implementation in community pharmacy settings according to health-system context, provider type and country income classification where sufficient evidence exists. As implementation science in antimicrobial stewardship continues to evolve, understanding how interventions can be adapted while maintaining implementation fidelity will be essential to achieving equitable and sustainable impact across diverse healthcare settings.

Beyond addressing evidence gaps, there is a critical need for sustained support for implementation research in resource-constrained settings, particularly in LMICs.^7^ This requires appropriate infrastructure, funding and local expertise to develop and evaluate contextually relevant interventions. More importantly, there is a need for collaboration between researchers in LMIC and high-income country (HIC), as those in HIC may not have a full understanding of the contextual challenges faced by healthcare providers in LMICs.^7^ Given that antimicrobial resistance transcends national borders, locally led but globally connected approaches are essential to reducing unnecessary antimicrobial prescribing.^8^

We appreciate the opportunity to clarify these points and thank the author for extending the discussion initiated by our review. We hope this exchange stimulates further implementation research to strengthen both diagnostic stewardship and antimicrobial stewardship across diverse primary care settings including community pharmacy globally.

## References

[1] Sorn V . Strengthening healthcare systems to prevent antimicrobial resistance in LMICs: it is time to act. J Glob Health 2026; 16: 03007. 10.7189/jogh.16.03007 PMC12981740 41818632

[2] Ximenes G, Saha SK, Dos Santos A et al A qualitative study exploring factors influencing antibiotic supply in community pharmacies in Timor-Leste. Int J Pharm Pract 2026; 10.1093/ijpp/riag071 42241440

[3] Belachew SA, Hall L, Selvey LA. Non-prescription dispensing of antibiotic agents among community drug retail outlets in Sub-Saharan African countries: a systematic review and meta-analysis. Antimicrob Resist Infect Contr 2021; 10: 13. 10.1186/s13756-020-00880-w PMC7807893 33446266

[4] Li J, Zhou P, Wang J et al Worldwide dispensing of non-prescription antibiotics in community pharmacies and associated factors: a mixed-methods systematic review. Lancet Infect Dis 2023; 23: e361–70. 10.1016/S1473-3099(23)00130-5 37105212

[5] Do NTT, Ta NTD, Tran NTH et al Point-of-care C-reactive protein testing to reduce inappropriate use of antibiotics for non-severe acute respiratory infections in Vietnamese primary health care: a randomised controlled trial. Lancet Glob Health 2016; 4: e633–41. 10.1016/S2214-109X(16)30142-5 PMC4985565 27495137

[6] Althaus T, Greer RC, Swe MMM et al Effect of point-of-care C-reactive protein testing on antibiotic prescription in febrile patients attending primary care in Thailand and Myanmar: an open-label, randomised, controlled trial. Lancet Glob Health 2019; 7: e119–31. 10.1016/S2214-109X(18)30444-3 PMC6293968 30554748

[7] Saha SK, Athan E. Promoting antibiotic pharmacotherapy in community pharmacies in low-and middle-income countries: why and how? J Pharm Pract 2026; 10.1093/ijpp/riag030 41770645

[8] Manesh A, Aslan AT, Mohan George M et al Global survey on the management of multidrug-resistant gram-negative Bacilli: divergence of practice between high-income countries and the rest of the world. Clin Infect Dis 2026; 82(Suppl 5): S105–15. 10.1093/cid/ciag025 42227055

